# Extra-Abdominal Desmoid Tumors Associated with Familial Adenomatous Polyposis

**DOI:** 10.1155/2012/726537

**Published:** 2012-06-03

**Authors:** George T. Calvert, Michael J. Monument, Randall W. Burt, Kevin B. Jones, R. Lor Randall

**Affiliations:** ^1^Department of Orthopaedics and Huntsman Cancer Institute, The University of Utah, Salt Lake City, UT 84112, USA; ^2^Sarcoma Services, Center for Children, Huntsman Cancer Institute, The University of Utah, Salt Lake City, UT 84112, USA; ^3^Department of Medicine and Huntsman Cancer Institute, The University of Utah, Salt Lake City, UT 84112, USA

## Abstract

Extra-abdominal desmoid tumors are a significant cause of morbidity in patients with familial adenomatous polyposis syndrome. Understanding of the basic biology and natural history of these tumors has increased substantially over the past decade. Accordingly, medical and surgical management of desmoid tumors has also evolved. This paper analyzes recent evidence pertaining to the epidemiology, molecular biology, histopathology, screening, and treatment of extra-abdominal desmoid tumors associated with familial adenomatous polyposis syndrome.

## 1. Introduction

 Desmoid tumors (DTs), also known as aggressive fibromatosis, are fibroblastic neoplasms which are often locally aggressive but lack metastatic potential. They may occur sporadically or in association with familial adenomatous polyposis (FAP) syndrome. Among individuals with FAP, desmoids most frequently occur in intra-abdominal and abdominal wall locations with most arising from the peritoneum. These abdominal desmoids range in severity from indolent, asymptomatic lesions to highly invasive, sometimes fatal tumors. Although less common than abdominal desmoids and very rarely fatal, extra-abdominal desmoids are also a significant cause of morbidity in this population. This paper will review recent developments in the diagnosis, screening, treatment, and prognosis of FAP-associated extra-abdominal DTs.

## 2. Epidemiology of FAP-Associated Desmoid Tumors

 The overall incidence of DTs has frequently been quoted at 2–4 per million people per year [1, 2]. This estimate is derived from a 1986 Finnish study which used the pathologic records of several regional hospitals and their known catchment area populations to calculate an incidence figure [3]. Recently, the Dutch national pathology database was analyzed, and 519 total desmoid cases in patients over the age of ten were identified from 1999 to 2009. There were 480 sporadic DTs and 39 FAP-DTs. The annual incidence was 3.7 per million overall [4] consistent with the earlier Finnish study. The same nationwide study from The Netherlands identified 1400 patients over the age of ten with FAP during the 1999 to 2009 period. FAP-associated DTs (FAP-DTs) made up 7.5% of all DTs, and the relative risk of an FAP patient developing a DT was over 800-fold higher than the general population [4]. The Dutch study was limited by the use of pathologic specimens as many DTs may be identified based upon history, physical exam, and imaging but not biopsied or surgically excised especially in the FAP cohort. Additionally, some individuals with sporadic DTs may have had as yet undiagnosed FAP. Therefore, FAP-DTs likely constitute more than 7.5% of all DTs.

 A 1994 study of the Johns Hopkins Polyposis Registry found that 10% (83/825) of FAP patients had desmoids, and their relative risk of DTs was 852-fold higher than the general population [5]. A study of Mayo Clinic data from 1976 to 1999 identified 447 desmoid patients of whom 70 (15.7%) had FAP [6]. In all of the previously mentioned studies, intra-abdominal and abdominal wall desmoids predominated in the FAP cohorts whereas extra-abdominal desmoids were most common among sporadic cases. The sites of extra-abdominal DTs (head and neck, trunk exclusive of abdominal wall, and extremity) do not appear to vary between the sporadic and FAP-associated desmoid cohorts. Other consistent demographic findings include younger age at DT presentation among FAP patients, history of abdominal surgery in abdominal DTs, and reduced female predominance of DTs among individuals with FAP [4–7]. Although females develop DTs more frequently than males in both FAP- and non-FAP-associated disease, the sex predominance is less in the FAP cohort.  Table 1 summarizes the known risk factors for DT development in FAP patients based upon the previous cited studies.

## 3. Desmoid Histology, Cytogenetics, and Immunohistochemistry

 Desmoids usually present grossly as firm, white tumors with a coarse, trabeculated surface. They may appear to be scar-like and encapsulated which belies their infiltrative behavior at the microscopic level. Histologic analysis reveals bland spindle-shaped cells in a collagenous stroma containing blood vessels [8]. The cells lack atypia, but the mitotic rate is variable [8]. Sporadic and FAP-DTs are indistinguishable at the gross and microscopic levels. Cytogenetic analyses of DTs (both sporadic and FAP-associated) have shown trisomies of chromosomes 8 and 20 to be recurrent abnormalities [9]. Trisomy 8 was found to correlate with recurrence in two separate studies [9, 10]. Immunohistological staining of DTs is positive for vimentin and variably positive for muscle and smooth muscle markers [8]. A study of 116 DT samples (both sporadic and FAP specimens) found only 7 estrogen receptor-beta-positive tumors, one C-KIT-positive tumor, and no HER2 or estrogen receptor-alpha-positive tumors [11]. A subsequent study of 40 desmoids using different immunohistological techniques found some degree of estrogen receptor beta expression in all samples whereas estrogen receptor alpha expression was absent in all samples [12].

## 4. Desmoids and the APC Gene Pathway

**Figure 1:**
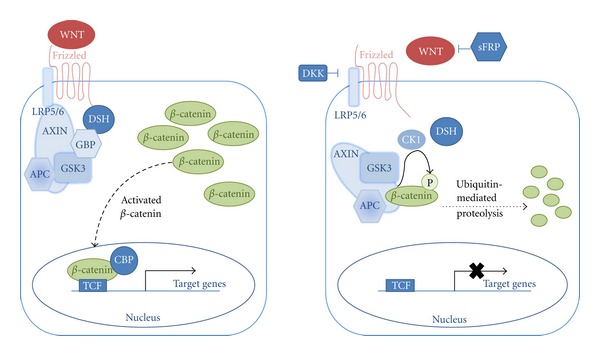
Model of the WNT/APC/beta-catenin pathway (Adapted from Moon et al. [18].)

 Mutation of the tumor suppressor Adenomatous Polyposis Coli (APC) gene was identified as the cause of FAP in 1991 by two different groups working independently [13–16]. The APC gene is located on the long arm of chromosome 5 (5q21); its product has been implicated in a wide variety of cellular processes including cell migration, cell adhesion, chromosome segregation, spindle assembly, apoptosis, and neuronal differentiation [17]. Despite these many roles, the classical function of APC in neopalsia is inhibition of the WNT signaling pathway. WNTs are a family of secreted glycoproteins which act as short range ligands in cell signaling. Binding of WNT on the cell surface upregulates the accumulation of beta-catenin in the cytoplasm, and the beta-catenin molecules subsequently move to the nucleus and activate WNT pathway transcription factors [18]. The APC gene product, located in the cytoplasm, forms a molecular complex with Glycogen Synthase Kinase 3 (GSK3) and Axin which in turn binds beta-catenin leading to its subsequent degradation [19]. The APC pathway is summarized in Figure 1.

 Both sporadic and FAP-DTs have been analyzed for APC and beta-catenin mutations. As expected, most FAP-DTs show a second somatic mutation of the APC gene [20]. However, the secondary somatic mutations of the FAP-DTs have been shown to differ consistently from the secondary somatic mutations in the colonic polyps from the same individuals [21]. APC mutations are infrequently found in sporadic DTs [22] which more frequently demonstrate beta-catenin mutations [23, 24].

## 5. Genotype Phenotype Correlations in FAP-Associated Desmoids

 The correlation of genotype with phenotype in FAP-DTs may permit more efficient screening strategies, improved treatment regimens, and ultimately targeted therapy of the disease. A variant of FAP, termed hereditary desmoid disease was first described by Eccles et al. in 1996 [25]. They reported 100% penetrance of desmoid tumors in a three-generation kindred with a mutation in the extreme 3′ end of the APC gene [25]. DTs in this kindred had both extra- and intra-abdominal involvement. Subsequently, Couture et al. reported a large French-Canadian kindred with a similar phenotype and extreme 3′ mutation of the APC gene [26]. This kindred had extensive desmoid disease and attenuated colonic polyp formation in contrast to classic FAP. These authors further demonstrated that desmoid tissue from a member of the kindred had elevated beta-catenin levels [26]. Prior studies of the secondary somatic mutations which occur in FAP colon polyps revealed that the type and location of the somatic mutation were nonrandom and at least partially determined by the location of the germ-line mutation [21, 27]. The APC gene product contains seven 20 amino acid beta-catenin degradation repeats (AARs). These repeat segments permit binding of beta-catenin leading to its ultimate degradation. The “just right” model of FAP tumorigenesis proposes that there is an ideal level of beta-catenin binding suitable for polyp progression to colon cancer, and selective pressure results in nonrandom selection of somatic mutations with the appropriate number of AARs [27]. Analysis of FAP-DTs by Latchford et al. revealed that 87% (26/30) of tumors had one allele with no AARs and preferentially retained a total of two AARs 57% (17/30) [28]. These authors suggested that specific levels of beta-catenin activity are required by the different tumor types with desmoids preferentially requiring two AAR segments. A large Japanese study (86 colorectal tumors, 40 extracolonic tumors) identified similar associations between AARs and phenotype. With respect to FAP-DTs, 5 of 6 were found to have two AARs in the Japanese study [29].

 Development of desmoids among individuals with FAP has been correlated with specific mutations. Early studies with small numbers of FAP-DTs suggested that mutations in these patients tended to occur at the 3′ end of the gene [30, 31]. A 2001 study from Hereditary Colorectal Tumor Registry in Milan analyzed 809 FAP patients of which 107 (11.9%) developed DTs including 59 extraabdominal cases [32]. These authors found a 12-fold increased risk of DT when the APC mutation occurred beyond codon 1444 as compared with upstream mutations [32]. In a multivariate analysis, these authors determined that genotype was the strongest predictor of desmoid development [32]. A 2007 review of the world literature on APC genotype/phenotype correlation identified ten articles with data on FAP-DTs. The reviewers concluded that patients with APC mutations downstream of codon 1400 were at increased risk of desmoid development [33]. More recently, genotype data have been incorporated into a desmoid risk scoring system for FAP patients. Female sex, presence of other extracolonic manifestations, a relative with a DT, and genotype were the risk factors considered [34]. The authors utilized the risks identified using this system to guide surgical management. They advocated use of antiadhesion material, sulindac prophylaxis, and minimally invasive techniques in patients at increased risk of desmoid formation [34].

## 6. Gene Expression Profiles of FAP-Associated Desmoids

 APC is a large protein with numerous binding sites and multiple putative functions. Gene expression profiling is one strategy which has been used to better understand the complex downstream effects of APC mutations. A critical factor in gene expression profiling is determination of which tissues should be compared because genes can only be up- or downregulated with respect to a reference specimen. With reference to DTs, numerous tissue samples have been studied including FAP-DTs, sporadic DTs, banked reference fibrous tissue, fibrous tissue from the same patient, adenomatous tissue from the same FAP patient, and many other banked histologic specimens. The technical aspects of each study are beyond the scope of this paper, but some notable findings merit discussion. The first desmoid gene expression profile study (2004) compared 12 sporadic DTs with banked normal fibrous tissue. Notably, the study identified two distinct groups within the 12 patients based upon gene expression, but no obvious clinical correlations were evident [35]. A 2006 study analyzed four tumors (2 with APC mutations, 2 with beta-catenin mutations) using normal fibrous tissue from the same patients as control. Sixty-nine differentially expressed genes were identified, of which 33 were upregulated and 36 were downregulated [36]. Interestingly, no differences in the profiles of the APC and beta-catenin tissues were identified. The authors additionally confirmed consistent downregulation of insulin-like growth factor-binding protein 6 using reverse transcriptase PCR and Northern blot assays [36].

 A study comparing desmoid samples (both sporadic and FAP associated) with nodular fasciitis was performed using 33 DTs and 11 nodular fasciitis specimens. Hierarchical clustering revealed distinct gene expression signatures between the two groups [37]. The authors concluded that this technology may be useful in diagnostically challenging cases. Gene expression profiling may also be of prognostic value as demonstrated by a 2007 study which found that elevated beta-catenin and p53 expression correlated with local recurrence in a retrospective analysis of 37 DTs (sporadic versus FAP not specified) [38]. A recent study reported the results of array comparative genomic hybridization analysis of 196 DTs (only 5% were FAP-DTs) [39]. Four recurrent chromosomal abnormalities were identified: loss of 6q, loss of 5q, gain of 20q, and gain of chromosome 8 [39]. Loss of 5q is likely explained by APC localization to this region. The other gains and losses suggest avenues of future investigation.

 A 2011 study compared sporadic and FAP-DTs using array comparative genomic hybridization analysis [40]. The authors analyzed 17 FAP-DTs and 38 sporadic DTs. They found more copy number abnormalities among the FAP-DTs than the sporadic DTs. Loss of 6q was common to both sporadic and FAP-DTs, and the authors believed that further study of genes in this region may help elucidate desmoid tumorigenesis [40]. They noted that several known or putative tumors suppressor genes including *ANKRD6*, *BACH2*, *MAP3K7/TAK1*, *EPHA7*, and *NLBP/KIAA0776* reside in this region. As yet, none of these putative tumor suppressors have been correlated with the downregulated genes identified in the previously discussed gene expression profile studies.

 Another application of gene expression profiling is analysis of treatment response. A 2010 report compared a FAP-DT human cell line with a sporadic DT human cell line using microarray analysis [41]. Doxorubicin-treated cells from each line were compared with each other and their untreated controls. Separate *in vitro* assays had already shown that the FAP-DT cell line demonstrated greater doxorubicin resistance than the sporadic DT cell line [41]. The gene expression profiles of the treated cells differed in that the pro-survival genes *netrin 1 *and *tumor necrosis factor receptor superfamily member 10c* were upregulated in the treated FAP-DT line and the proapoptotic gene *forkhead box L2 *was upregulated in the treated sporadic DT line [41]. Although this study was preliminary and *in vitro*, gene expression profiling may ultimately be applicable to prediction of response to treatment in humans.

## 7. Desmoid Cell of Origin

 As recently as 2000, debate existed as to whether desmoids were neoplastic or reactive. A 2000 study by Middleton et al. demonstrated that FAP-DTs were monoclonal [42]. The authors derived a clonality ratio by assessing X chromosome inactivation in desmoid samples from 12 female patients. Although it is now generally agreed that desmoids, both sporadic and FAP associated, are neoplastic, the cell of origin has yet to be identified. Recent animal studies suggest that mesenchymal stem cells (MSCs) are likely candidates and at minimum contribute to tumor development. Wu et al. recently demonstrated that MSCs and desmoids had similar gene expression profiles, and mice deficient in MSCs but prone to desmoids (mice with an APC mutation and deficient MSC production) developed fewer desmoid tumors while colonic tumor rates were uneffected [43]. In fact, desmoid development was directly proportional to the number of MSCs present. Additionally, MSCs with the APC mutation from heterozygote *APC^wt/1638N^* mice produced DTs when transplanted to immunodeficient mice, but MSCs without the mutation did not. Furthermore, they found that MSCs from mice with inducible expression of beta-catenin (*Catnb^tm2kem^* mice) could also induce desmoid-like tumors when transplanted to immunodeficient mice. Finally, they showed that these tumors were clonally derived from the donor MSCs with use of a green florescent protein tag [43].

 A 2012 study has further defined the role of mesenchymal stem cells in FAP-DTs using human tissue. Carothers et al. analyzed 16 human desmoid specimens and using immunohistochemistry found that desmoid tissue expressed MSC markers but surrounding normal tissue did not [44]. They next developed a primary desmoid cell line from the human desmoid tissue. These cells had an immunohistochemical profile consistent with MSC, and the cells were able to differentiate into chondrocytes, osteocytes, and adipocytes confirming that they are MSCs [44]. These human desmoids-derived MSCs were found to have elevated beta-catenin in their nuclei (similar to desmoid tissue) and demonstrated upregulation of the Notch and Hedgehog pathways [44].

 The aforementioned studies do not definitively prove that MSCs are the cell of origin in FAP-DTs, but they at a minimum demonstrate the importance of MSCs in desmoid development. The association between desmoid development and surgical wound healing in patients with FAP has long been established [45]. Presence of extra-abdominal and abdominal wall DTs increases the risk of intra-abdominal DT development at the time of prophylactic colon resection [46]. A recent case report analyzed the individual tumor mutations of a FAP patient with multiple recurrences at the same surgical site. Interestingly, different APC mutations were identified in the “recurrent” tumors suggesting that these were in fact new clonal populations and not true recurrences [47]. Based upon the previously noted findings, one can postulate a model in which secondary somatic mutations develop in the MSC rich wound healing environment of FAP patients. This model fits well with the known development of desmoids after surgical or incidental trauma in the FAP population.

## 8. FAP Screening and Treatment Guidelines in relation to Desmoid Treatment

 Physicians specializing in the treatment of sarcomas will rarely be the first to diagnose FAP because desmoids in these patients most frequently occur after gastrointestinal manifestations of the disease are evident. Additionally, many kindreds have been extensively tested, and affected family members are frequently diagnosed early in childhood. However, de novo mutations may occur, and individuals with FAP may still initially present with extracolonic manifestations such as desmoids. A meta-analysis of desmoid risk among FAP patients identified family history of DT, APC mutation 3′ to codon1399, previous abdominal surgery, and female sex to be significant risk factors for DTs [48]. The same analysis found that 80% of FAP-DTs occur before age 40 [48]. Two other studies have noted that FAP-DTs present at a younger age in females than males [45, 49]. Practitioners should therefore suspect FAP in patients with a family history of desmoids and in young patients presenting with desmoids. Referral to gastroenterologists, geneticists, and colon and rectal surgeons experienced in FAP care is critical if the diagnosis is suspected. Many cancer centers have well established multidisciplinary groups and polyposis registries. A 2006 review of screening guidelines recommended careful postcolectomy follow-up to asses for desmoids as early intervention has anecdotally improved outcome for some [50]. Practical surveillance measures for all FAP patients include asking them about new masses and examining their body surface for tumors at each visit.

 Other extracolonic manifestations of FAP should be considered by the clinician treating FAP-DTs. Gastric polyps were found in 88% of FAP in a 2008 study of 75 consecutive FAP patients, and gastric cancer rates are increased in this population [51]. Duodenal and papillary adenomas occur in 50–90% of FAP patients, and there is an overall 5% lifetime risk of duodenal cancer in FAP patients [52, 53]. Routine surveillance of the upper gastrointestinal tract with endoscopy is therefore recommended [53]. APC is a tumor suppressor gene and is associated with other cancers including papillary thyroid carcinoma, hepatoblastoma, medulloblastoma and other brain tumors, and pancreatic cancer [54]. The associated cancer risks are low (1-2% for each diagnosis) compared with the 100% risk of colon cancer in untreated FAP [33, 54]. However, these associated tumors (except pancreatic cancer) tend to occur at a young age, often before gastrointestinal manifestations develop. This fact further emphasizes the importance of genetic testing of at-risk individuals. Nonmalignant FAP associations include adrenal tumors, osteomas, congenital hypertrophy of the retinal pigment epithelium (CHRPE), and dental abnormalities [33, 54]. Most of these nonmalignant entities do not cause significant morbidity, and as previously noted DTs are the most clinically significant nonmalignant extracolonic manifestation of the disease.  Table 2 summarizes the extra-colonic manifestations of FAP.

## 9. Evolving Trends in the Surgical Management of FAP-DTs

 The surgical treatment paradigm for DTs in general has changed substantially over the past decade. Overall, a less aggressive surgical approach has been adopted by many centers. In 1989, a large series (131 patients, both sporadic and FAP-DT) from Memorial Sloane-Kettering was published detailing desmoid cases at the institution from 1965 to 1984. Adequacy of surgical margin was found to be the single most important factor in successful treatment of desmoids [55]. The authors concluded that “aggressive resection in an effort to obtain as wide a margin as possible is clearly the single most important determinant of successful outcome” [55]. A Mayo Clinic series reporting extra-abdominal desmoid cases from 1981 to 1989 similarly found a high local recurrence rate (9/19) in patients with microscopic residual disease [56]. In 1999, another report (105 patients with primary desmoid disease, both sporadic and FAP-DT) from Memorial Sloan-Kettering covering the years 1982–1997 did not find positive microscopic margin to be predictive of local recurrence [57]. These later authors recommended against excessively morbid resections in an effort to obtain wide margins. In 2003, Gronchi et al. reported a series of 203 consecutive desmoid patients treated over 35 years at a single institution. They found that microscopic positive margins did not adversely affect recurrence rates for primary disease [58]. They recommended function sparing surgery and resection of all macroscopic disease but avoidance of heroic attempts at obtaining negative microscopic margins. A smaller series from the United Kingdom reported the results of surgery for 32 FAP-DTs including 16 intra-abdominal, 12 abdominal, and 4 extra-abdominal tumors treated from 1994 to 2004. In contrast to some prior reports of abdominal desmoids in FAP patients, they had no desmoids-related mortalities and only one patient required long-term parenteral nutrition [59]. These authors noted that they had a high threshold for surgery, and that most intra-abdominal desmoids at their institution were treated conservatively.

 Even more recently, several authors have begun advocating a *wait and see* approach to DTs as it has been recognized that many DTs undergo a prolonged stable phase or even spontaneous regression. A 1998 article from this journal reported a series of 17 patients treated nonoperatively, all of whom had an interval of at least six months without disease progression [60]. 

Subsequently, a French report identified a subgroup of patients who did well with a *wait and see* approach. Only 23 patients were included in the nonoperative group, and there were no strict inclusion criteria [61]. A subsequent, larger study analyzed the results of a routine front-line conservative approach used to treat both primary and recurrent desmoids at two institutions [63]. Seventy-four primary and 68 recurrent tumors were studied. Eighty-three received no intervention, and 59 received medical therapy. Overall progression-free survival was 64% at 3 years and 53% at 5 years. There was not a statistically significant difference in progression free survival between the no intervention and the medically treated groups [63]. The authors did not believe that subsequent surgery was compromised by delay in the patients who progressed. More recently, a study was performed to identify factors associated with progression free survival. In a multivariate analysis of 426 sporadic desmoid tumors, age less than 37, extremity location, and size greater than 7 cm were associated with progression [65]. Notably, the authors could not determine how to use this information with respect to surgery versus *wait and see*. One could argue that DTs at high risk of progression should be resected early because conservative treatment is more likely to fail. On the contrary, perhaps the high-risk group should be observed because they may be more biologically aggressive and therefore more likely to recur after surgery. This cannot be answered without prospective data.

 Most of the aforementioned studies included few if any FAP-DTs. There are no studies which show that FAP associated extra-abdominal desmoids behave differently than their sporadic counterparts with respect to surgical management of primary disease. As previously discussed, FAP-DTs may occur after surgery and trauma. This phenomenon is presumably related to the wound healing environment in the setting of germ-line APC mutations. A conservative approach to intra-abdominal desmoids has long been recommended due to the high morbidity and even mortality noted in many early studies [64, 66]. Modern studies of FAP-DTs have shown that resection is surgically safe but recurrence rates remain high. Consensus for first-line conservative management is growing [63–65]. The studies referenced in this section are summarized in Table 3.

## 10. Medical Treatment of FAP-Associated Extra-Abdominal Desmoids

 Current first-line medical management includes antihormonal therapy (specifically tamoxifen) and nonsteroidal anti-inflammatory drugs (NSAIDs, specifically sulindac, indomethacin, and more recently celecoxib) [62]. A recent review of antiestrogen therapy for DTs found that approximately half of patients respond, and response does not appear to correlate with estrogen receptor status [67]. Furthermore, the desmoid location and FAP status of the patient do not appear to influence the response [67]. NSAIDs have shown efficacy against desmoids in numerous studies, but the mechanism of action of these agents is even less clear than that of antidestrogen therapies [68]. A mouse model of APC-associated desmoid tumors was found to have elevated levels of cyclooxygenase-2, and mice treated with a cyclooxygenase-2 inhibitor had decreased desmoid tumor size [69]. There are little human data corroborating the effects of prostaglandins and prostaglandin inhibition on DTs.

 Multiple chemotherapeutic agents have shown efficacy against desmoids including doxorubicin, methotrexate plus vinblastine, cyclophosphamide plus doxorubicin, and VAC (vincristine, actinomycin-D, cyclophosphamide) [68, 70]. Interferon alpha has also been used singly and in combination with some of the aforementioned cytotoxic agents [68]. More recently, targeted biologic agents have been added to the desmoid treatment armamentarium. Two phase 2 trials have reported efficacy of imatinib, a tyrosine kinase inhibitor, in the treatment of desmoids [71, 72]. As previously mentioned, *C-KIT* expression is lacking in most DTs. Analysis of 124 DTs from 85 patients found that *PDGF alpha* and *PDGF receptor alpha* were expressed in all tumors, but *PDGF beta* and *PDGF receptor beta* were not expressed [73]. The same authors failed to identify PDGF receptor mutations in 14 analyzed specimens [73]. These data suggest that imatinib's efficacy against desmoids results from a mechanism other than direct inhibition of these known tyrosine kinase protooncogenes. Another tyrosine kinase inhibitor, sorafenib, has also shown efficacy against desmoids in a smaller single-institution trial [74]. Finally, a clinical trial (NCT01265030) of the mammalian target of rapamycin (mTOR) inhibitor, sirolimus, for the treatment of desmoids in children and young adults was opened in 2010. The large number of agents used for DTs clearly indicates that presently there is lack of consensus with respect to medical management of this condition.

## 11. Conclusion

 Understanding of the epidemiology, genetics, molecular and cellular biology, pathophysiology, and treatment of FAP related desmoid tumors has improved substantially over the past decade. Despite these improvements, DTs remain a major cause of morbidity in the FAP population. A more conservative surgical approach is presently advocated by many oncologic surgeons. Medical management is attempted first for most abdominal DTs, and a *wait and see* approach is undertaken for many extra-abdominal DTs. Surgical goals and techniques are now often less aggressive than in the past. Recent studies have implicated mesenchymal stem cells as critical components of desmoid development. Gene expression profiling has shown promise in elucidating downstream elements of the WNT/APC/beta catenin pathway. Future progress in treatment will likely depend upon continued advances in understanding of basic desmoid biology and the development of additional targeted therapies for the treatment of refractory cases.

## Figures and Tables

**Table 1:** Demographic risk factors for desmoid development among FAP patients.

Risk Factor	Reference
Younger age	[4]
Male sex	[4]
Intra-abdominal location	[4]
Abdominal wall Location	[4]
Mutation 3′ of codon 1444	[7]
Previous abdominal surgery	[7]

**Table 2:** Extracolonic FAP manifestation (Neiuwenhuis [33] and Groen [54]).

Extracolonic manifestation	Prevalence in FAP patients
CHRPE	70–75%
Osteomas and dental abnormalities	70–90%
Upper GI tumors	50–90%
Epidermoid cysts and lipomas	25–50%
Desmoid tumors	15–20%
Adrenal tumors	7–13%
Papillary thyroid cancer	1-2%
Hepatoblastoma	1-2%
Brain Tumors (Medulloblastoma and others)	1-2%
Pancreatic Cancer	1%

**Table 3:** Surgical and nonoperative outcomes from selected studies.

References	Subjects	Anatomic site	Presentation	Intervention	Surgical margins	Follow-up	Outcomes
Posner et al. [55]	Retrospective review (*n* = 131)	Extra-abdominal	Primary (*n* = 131)	Surgery (*n* = 131)	Not reported	Median F/U 88 months	36% LR Median time to LR: 15 months Negative microscopic margins predictive of EFS
Pritchard et al. [56]	Retrospective review (*n* = 50)	Extra-abdominal	Primary (*n* = 50)	Observation (*n* = 3) XRT (*n* = 3) Surgery (*n* = 34) Surgery + XRT (*n* = 10)	Negative (*n* = 15) Positive/marginal (*n* = 29)	Minimum F/U 48 months	Observation/XRT: 1/6 no progression Negative margins: 2/15 LR; 13/15 no LR Positive/marginal margins: 12/29 LR; 17/29 no LR
Merchant et al. [57]	Prospective cohort (*n* = 105)	Extra-abdominal	Primary (*n* = 105)	Surgery (*n* = 105) Adjuvant XRT (*n* = 31)	Negative (*n* = 58/105) Positive (*n* = 47/105)	Mean F/U 49 months	Negative margin: 14/58 LR (24%) Positive margin: 12/47 LR (26%) No difference in LR with adjuvant XRT
Gronchi et al. [58]	Retrospective review (*n* = 203)	Extra-abdominal	Primary (*n* = 128) Recurrent (*n* = 75)	Surgery (*n* = 203)	Negative margin (*n* = 146) Positive margin (*n* = 56)	Median F/U 135 months	De novo DT: 76% LR Recurrent DT: 59% LR Positive margins not predictive of recurrence for de novo DT
Latchford et al. [59]	Retrospective review (*n* = 20)	FAP associated Extra-abdominal and abdominal	Primary (*n* = 20) (32 tumors)	Surgery (*n* = 20) Medication (*n* = 19)	Not reported	Median F/U 60 months	42% LR in macroscopically complete resections No desmoid-related mortalities
Bonvalot et al. [61]	Retrospective review (*n* = 112)	Extra-abdominal	Primary (*n* = 112)	Nonoperative (*n* = 23) Observation (*n* = 11) Medication (*n* = 12) Surgery (*n* = 89) Medication (*n* = 9) Adjuvant XRT (*n* = 13)	Negative (*n* = 19/89) Positive (*n* = 70/89)	Median F/U 76 months	Nonoperative group: 14/23 stable disease Surgical resection: 57/89 LR (64%) Similar EFS with nonoperative treatment and negative margin surgical resection Tumor location and negative margin predictive of EFS
Stoeckle et al. [62]	Retrospective review (*n* = 106)	Extra-abdominal and abdominal	Primary (*n* = 69) Recurrent (*n* = 37)	Medication (*n* = 11) XRT (*n* = 23) Surgery (*n* = 92)	Negative (*n* = 22) Positive (*n* = 70)	Median F/U 129 months	Increased LR with recurrent disease Functional impairment correlates with number of surgeries Time to stable disease increased with number of surgeries
Fiore et al. [63]	Retrospective review (*n* = 142)	Extra-abdominal and intra-abdominal	Primary (*n* = 74) Recurrent (*n* = 68)	Observation (*n* = 83) Medication (*n* = 59)	N/A	Median F/U 33 months	Observation: 5 year PFS: 50% Medical therapy: 5 year PFS: 59%
Nieuwenhuis Vase [64]	Retrospective review (*n* = 78)	FAP associated Extra-abdominal and abdominal	Primary (*n* = 78)	Surgery (*n* = 49) Non-operative (*n* = 29)	Not reported	Median F/U 96 months	Abdominal DT: similar PFS with operative and non-operative therapy Extra-abdominal DT: PFS favors surgical resection
Salas et al. [65]	Retrospective review (*n* = 426) multicenter database	Extra-abdominal and abdominal	Primary (*n* = 426)	Observation (*n* = 27) Medication (*n* = 23) XRT (*n* = 6) Surgery (*n* = 370) Surgery + XRT (*n* = 37)	Negative margin (*n* = 111) Positive margin (*n* = 147) Unknown (*n* = 112)	Median F/U 54 months	Observation only: 78% stable/remission Negative margins: 44% LR; 64% no LR Positive margins: 67% progression; 33% no progression

EFS: event free survival; PFS: progression free survival; LR: local recurrence; F/U: follow-up; DT: Desmoid tumor; XRT: radiation therapy.
